# Gut microbiota-derived short-chain fatty acids in gallstones: mechanisms and intervention strategies

**DOI:** 10.3389/fmicb.2026.1897348

**Published:** 2026-08-31

**Authors:** Linghan Xue, Tingting Yu, Mengyao Jiang

**Affiliations:** 1 The Second Hospital of Hebei Medical University, Shijiazhuang, China; 2 Biliopancreatic Endoscopic Surgery Department, The Second Hospital of Hebei Medical University, Shijiazhuang, China; 3 Department of Basic Medical Sciences, Hebei Medical University, Shijiazhuang, China

**Keywords:** bile acid metabolism, biliary microbiota, gallstone, gut microbiota, short-chain fatty acids

## Abstract

Gallstones are a common digestive disease in humans. They may result in cholecystitis, cholangitis, and other complications, thereby causing serious harm to human health. Accumulating evidence suggests that gut dysbiosis is associated with gallstone formation. As key metabolites of the gut microbiota, short-chain fatty acids (SCFAs) may exert protective effects on gallstones through multiple mechanisms. These mechanisms include regulating lipid and bile acid metabolism, maintaining the intestinal barrier, modulating the microbial community structure, and reducing the risk of cholecystitis. However, a systematic synthesis of these mechanisms is still lacking. Therefore, this review provides a comprehensive overview of the research progress on this topic. It systematically mapped the metabolic pathways of core SCFA-producing bacteria and consolidated the current evidence for their potential anti-lithogenic effects. We also analyzed possible mechanisms by which SCFAs might suppress gallstone formation. Furthermore, clinical intervention strategies are proposed and critical knowledge gaps are identified. A deeper insight into the link between SCFAs, their producers, and gallstones may reveal novel preventive and therapeutic targets.

## 1 Introduction

Gallstone disease is a common digestive disorder, affecting 10–20% of the global population (Du et al., 2026). It frequently leads to cholecystitis, acute pancreatitis, and other complications, increasing the medical burden (Gupta et al., 2024). Gallstone formation is influenced by multiple factors, such as excessive cholesterol levels, poor gallbladder motility, and abnormal mucin secretion, and chronic cholecystitis persists throughout gallstone development (Sun et al., 2022; Bai X. et al., 2025; Ren et al., 2025). However, these mechanisms cannot fully account for cases without traditional risk factors, suggesting that additional factors play a role in gallstone formation.

Recent studies have shown that gut microbiota imbalance is associated with gallstones (Binda et al., 2022). Gut microbiota is the largest microecosystem in the human body. It participates in nutrient absorption, immune regulation, and metabolic balance (Olteanu et al., 2024). Among these metabolic activities, it breaks down dietary fiber to produce SCFAs, mainly acetate, propionate, and butyrate (de Vos et al., 2022). These metabolites act as signaling molecules and have been found to exert beneficial effects on inflammatory bowel disease, colorectal cancer, atherosclerosis, and gallstones (Haghikia et al., 2021; González et al., 2024; Ventura et al., 2024). Specifically in gallstones, SCFAs produced by the gut microbiota could act on the hepatobiliary system via the gut–liver axis. The gut–liver axis refers to the bidirectional connection between the intestine and its microbiota and the liver, established through the portal vein, biliary tract, and systemic circulation (Hsu and Schnabl, 2023). Through this axis, SCFAs may play protective roles by inhibiting cholesterol synthesis, regulating bile acid metabolism, maintaining the intestinal barrier, restoring gut flora, and alleviating cholecystitis (Ye et al., 2021; Chen et al., 2024; Mann et al., 2024; Sun et al., 2024). These findings suggest that SCFAs may be a potential target for the prevention and treatment of gallstones.

**Figure 1:**
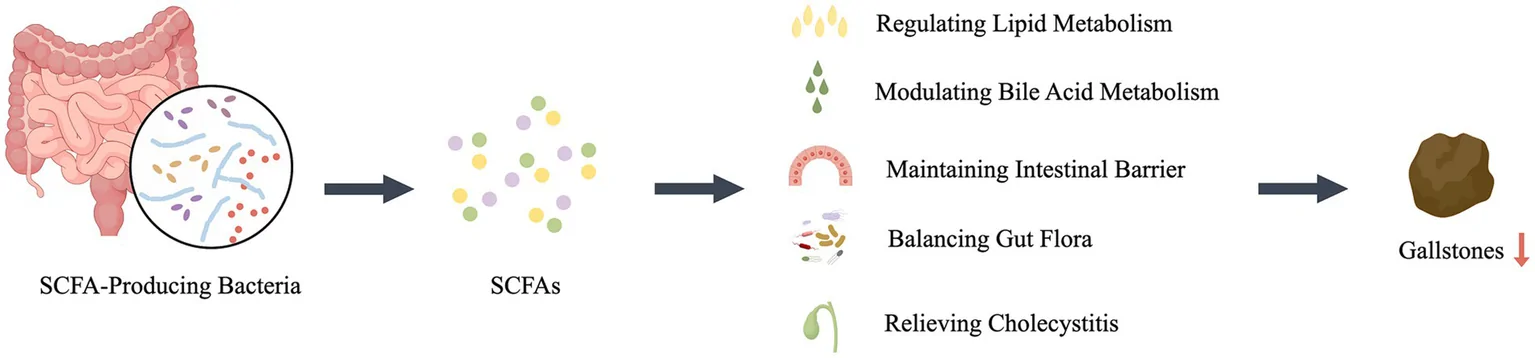
Schematic diagram illustrating potential protective effects of gut microbiota-derived short-chain fatty acids (SCFAs) via multiple mechanisms: SCFAs derived from intestinal SCFA-producing bacteria may attenuate gallstone formation via regulating lipid metabolism, modulating bile acid metabolism, maintaining the intestinal barrier, balancing gut flora, and relieving cholecystitis.

Although many studies have found a relationship between SCFAs and gallstones, few papers have systematically elucidated their interactions and underlying mechanisms. Therefore, this review focuses on this theme. It explores the metabolic characteristics of SCFA-producing bacteria and their associations with gallstones, while outlining the potential protective effects of SCFAs in five aspects: lipid metabolism, bile acid metabolism, intestinal barrier integrity, gut microbiota structure, and inflammation regulation. Table 1 presents a summary of the relevant literature. Intervention strategies and current research limitations are also discussed, providing theoretical support for the nonsurgical prevention and treatment of gallstones. Figure 1 illustrates the viewpoints and conceptual model of this review.

**Table 1:** Core literature summary table.

Section	References	Core findings	Study subjects	Limitations	Future directions
Gut bacteria producing SCFAs	Park et al. (2022)	Introduction of butyryl-CoA dehydrogenase and butyryl-CoA:acetate CoA-transferase genes from *Faecalibacterium prausnitzii* into *Escherichia coli* confirmed that butyryl-CoA dehydrogenase is a key rate-limiting enzyme for butyrate synthesis	*Escherichia coli* genetically engineered strain (*in vitro*)	Current research focuses mostly on a few genera (*Roseburia, Faecalibacterium prausnitzii, Bifidobacterium, Eubacterium hallii*), while the metabolic characteristics and ecological functions of other potential SCFA producers remain poorly understood; *in vitro* and animal studies predominate, and it is unclear how colonization patterns and cross-feeding networks of these bacteria in the human gut are regulated by diet and host factors	It should be clarified which other gut bacteria possess SCFA-producing capacity and their metabolic features; the conclusions from *in vitro* and animal experiments should be verified in humans; the effects of host factors such as diet, age, and medication on the colonization and interaction networks of SCFA-producing bacteria should be elucidated
	Shetty et al. (2020)	*Anaerobutyricum* and *Anaerostipes* can convert lactate and acetate into butyrate, revealing the molecular mechanisms of cross-feeding	Anaerobic gut bacteria (*in vitro*)		
	Li Q. et al. (2024)	*Eubacterium hallii* converts 1,2-propanediol into propionate via cobalamin-dependent glycerol/diol dehydratase	Carnivore gut microbiome genomic data		
	Nie et al. (2021)	*Roseburia intestinalis* produces butyrate via the butyryl-CoA:acetate CoA-transferase pathway, and regulates barrier homeostasis and immune cell function through butyrate and flagellin	Literature review		
	Bhattacharya et al. (2022)	*Roseburia hominis* and *Bifidobacterium adolescentis* exhibit cross-feeding on mannan-oligosaccharides; *Roseburia hominis* depends on exogenous acetate for butyrate production	Co-culture of *Roseburia hominis* and *Bifidobacterium adolescentis* (*in vitro*)		
	Modesto et al. (2021)	Describes enzymatic and genetic methods for detecting fructose-6-phosphate phosphoketolase activity in bifidobacteria; this enzyme is key to the bifid shunt	*Bifidobacterial* strains (*in vitro*)		
	Pan et al. (2022)	Polysaccharides enhance acetate output and reduce lactate secretion in *bifidobacteria*, providing more substrates for butyrate-producing bacteria	*Bifidobacterium* and polysaccharide fermentation system (*in vitro*)		
	de Vos et al. (2022)	Gut microbiota participates in host nutrient absorption, immune regulation, and metabolic balance through metabolites including SCFAs	Literature review		
	Culp and Goodman (2023)	Cross-feeding is the ecological basis for stable SCFA production; substrate dependencies among different species determine the composition of the SCFA profile	Literature review		
The association between SCFA-producing bacteria and gallstones
	Pichler et al. (2020)	Monosaccharides derived from mucin degradation can be utilized by Roseburia to support its growth and butyrate synthesis	Interaction between *Roseburia* and *Akkermansia muciniphila* (*in vitro*)	Observational studies are mostly cross-sectional and cannot determine whether microbiota changes are a cause or consequence of cholesterol gallstones; Mendelian randomization can suggest causality but cannot clarify mechanisms; heterogeneity across populations, regions, and detection methods is high, and there is a lack of consistent microbial markers; whether strains effective in animal models are equally effective in humans remains unproven	It should be determined whether microbiota changes are a cause or consequence of cholesterol gallstones; the specific mechanisms behind the causal associations suggested by Mendelian randomization should be elucidated; uniform microbial markers comparable across populations should be established; the efficacy of strains effective in animal models should be validated in humans
	Soto-Martin et al. (2020)	Many *Roseburia* strains cannot synthesize thiamine or folate and rely on exogenous supply; their abundance may be influenced by cofactors provided by other microbes or diet	Butyrate-producing bacteria (*in vitro*)		
	Shin et al. (2025)	In *Faecalibacterium prausnitzii*, 3-hydroxybutyryl-CoA dehydrogenase catalyzes a key redox step; its activity is regulated by pH, temperature, and NADH/NAD^+^ balance	*Faecalibacterium prausnitzii* L2-6 (*in vitro*)		
	Lindstad et al. (2021)	*Faecalibacterium prausnitzii* can hydrolyze *β*-mannan released by *Bacteroides* into oligosaccharides to meet its metabolic needs	*Faecalibacterium prausnitzii* (*in vitro*)		
	Onodera et al. (2025)	*Faecalibacterium prausnitzii* utilizes human milk oligosaccharides secreted by *Bifidobacterium* for metabolism	Cross-feeding between *Faecalibacterium prausnitzii* and *Bifidobacterium* (*in vitro*)			Chen et al. (2021)	SCFA-producing bacteria such as *Lachnospiraceae*_UCG-008 are significantly reduced in cholesterol gallstone model mice, correlating with changes in liver metabolites	C57BL/6 mice
	Georgescu et al. (2022)	*Faecalibacterium prausnitzii* and *Dorea* abundances are lower in middle-aged and elderly gallstone patients	Middle-aged and elderly gallstone patients		
	Chang et al. (2024)	*Prevotellaceae*_2 and *Ruminococcus*_2 are decreased in gallstone patients, with reduced fecal butyrate levels	Taiwanese gallstone patients		
	Lyu et al. (2021)	Bile of patients with primary choledocholithiasis shows significantly lower levels of *Roseburia* and *Butyrivibrio* compared to controls	Patients with primary choledocholithiasis		
	Li W. et al. (2023)	Mendelian randomization analysis suggests that genera such as *Phascolarctobacterium* are causally associated with reduced gallstone risk	Population dataset		

	Liu X. et al. (2023)	*Holdemania* and *Lachnospiraceae* UCG_010 are associated with reduced cholesterol gallstone risk	Population dataset		
	Yin and Zhang (2024)	Mendelian randomization confirms a genetic association between the genus *Butyricoccus* and reduced gallstone risk	Population dataset		
	Zhao J. et al. (2024)	*Faecalibacterium*_2 is associated with reduced cholesterol gallstone risk, whereas *Faecalibacterium*_3 is associated with increased risk, possibly due to differences in bile salt hydrolase activity	Population dataset		
	Takeda et al. (1983)	*Clostridium butyricum* reduces the incidence of experimental cholesterol gallstones and dissolves already formed stones	animal model of cholesterol gallstones		
	Li G. et al. (2023)	*Clostridium butyricum* exerts anti-cholesterol-gallstone effects by modulating gut microbiota structure and bile acid metabolism	C57BL/6 mice		
	Ye et al. (2022)	*Lactobacillus* regulates bile acid synthesis via the FXR–FGF15 axis, reducing biliary cholesterol saturation and inhibiting cholesterol gallstone formation	C57BL/6 mice		
	Che et al. (2025)	*Lactobacillus rhamnosus* B16 regulates lipid metabolism homeostasis through acetate production, indirectly inhibiting cholesterol gallstone formation	High-fat diet-induced mouse model		
	Wang et al. (2020)	Gut dysbiosis in gallstone patients affects bile acid metabolism, and reduced SCFA producers are associated with stone formation	Gallstone patients		
Grigor’eva and Romanova (2020)	Gut dysbiosis, characterized by expansion of pathogens and reduction of beneficial bacteria, is closely associated with cholesterol gallstone formation	Literature review			
Anti-gallstone mechanisms of gut microbiota-derived SCFAs
	Binda et al. (2022)	Gut microbiota imbalance is a key contributor to cholesterol gallstones, providing a broad background for the association between SCFA producers and gallstones	Literature review	Studies on the anti-stone mechanisms of SCFAs mostly focus on single dimensions; the interconnections and synergistic effects among the five dimensions remain unclear; the functional differences and interactions among different SCFAs are poorly understood	The interconnections and synergistic effects among the five anti-stone dimensions should be clarified; the functional differences and interactions among different SCFAs should be systematically compared	Ye et al. (2021)	Sodium butyrate alleviates cholesterol gallstones by regulating bile acid metabolism; SCFAs show region-specific modulation of FXR in the cecum and ileum	C57BL/6 mice
	Xu et al. (2022)	SCFAs exert an inhibitory effect on ileal FXR	Hypercholesterolemic hamsters		
	Cai et al. (2022)	SCFAs suppress conversion of primary bile acids to secondary bile acids, lowering the hydrophobicity index of the bile acid pool and reducing cholesterol precipitation	Literature review		
	Hays et al. (2024)	Butyrate upregulates tight junction proteins via STAT3/Sp1 pathways, enhancing the intestinal barrier	Literature review		
	Xu et al. (2023)	Butyrate enhances tight junction assembly through AMPK/Akt pathways, maintaining intestinal barrier integrity	Caco-2 cells (*in vitro*), mouse colonic tissue		
	Liang et al. (2022)	Butyrate activates the MUC2 promoter and stimulates goblet cells to secrete MUC2, thickening the intestinal mucus layer and reinforcing the chemical barrier	Intestinal organoids (*in vitro*), mouse model		
	Firrman et al. (2022)	When pH drops, *Lachnospiracea*e an*Lactobacillus* increase while *Escherichia coli* and *Shigella* decline sharply, optimizing the gut microenvironment	M-SHIME fermentation model (*in vitro*)		
	Liu T. et al. (2023)	SCFAs activate GPR43 to stimulate antimicrobial peptide release, directly eliminating pathogens and remodeling the gut microbiota	Literature review		

Al-Roub et al. (2021)	Acetate reduces TNF-α-induced MCP-1 production via GPR43, suppressing inflammatory cascades	Human monocytic cell line THP-1 (*in vitro*)		

Zhong et al. (2025)	Butyrate inhibits HMGCR activity and downregulates cholesterol synthesis gene expression, reducing cholesterol synthesis	Literature review		

Haghikia et al. (2021)	Propionate restricts hepatic cholesterol synthesis through immune-dependent pathways	ApoE^−^/^−^ mice, macrophages (*in vitro*)		

Chang et al. (2026)	Acetate combined with propionate has a synergistic lipid-lowering effect	Pig intestinal inflammation model		

Ornelas et al. (2023)	Butyrate maintains HIF-1α stability through β-oxidation-mediated oxygen consumption, indirectly strengthening the intestinal barrier	Chemically synthesized HIF-1α stabilizer (*in vitro*), cell experiments (*in vitro*)		

Yu J. et al. (2024)	LPS in bile promotes neutrophil extracellular trap-induced gallstone formation; SCFAs may reduce LPS translocation by maintaining barrier function	Gallstone mouse model		

## 2 Gut bacteria producing SCFAs

Gut microbial metabolism underlies SCFA production. SCFAs are volatile fatty acids with 2–6 carbons, primarily acetate, propionate, and butyrate. In healthy adults, they are produced at a molar ratio of approximately 60:20:20, with acetate predominating (Lange et al., 2023). Acetate concentrations range from 42–84 mM in the distal gut, 375 μM in the portal vein, 148 μM in the hepatic vein, 100–200 μM in systemic circulation, and 79 μM in peripheral fluids. Butyrate is present at 14–28 mM in the colonic lumen and 1–15 μM in peripheral fluids, while propionate shows a similar range of 14–28 mM in the proximal colon and 1–15 μM in peripheral fluids (Sankarganesh et al., 2025). In patients with gallstones, fecal SCFA levels are altered, characterized by reduced total output and a lower butyrate proportion, reflecting gut dysbiosis and impaired fermentation (Chang et al., 2024). Stable SCFA production depends on cooperation among different bacteria (Culp and Goodman, 2023). This section introduces key bacteria in the SCFA production network. These include *Faecalibacterium prausnitzii, Roseburia, Bifidobacterium, Eubacterium hallii,* and others. Table 2 presents the metabolic characteristics and cross-feeding partners of different SCFA-producing bacteria.

**Table 2:** Metabolic traits and syntrophic relationships of intestinal SCFA-producing bacteria.

Strain (representative species)	Products	Substrates	Metabolic pathways (key enzymes)	Cross-feeding partners	Cofactors	References
*Akkermansia muciniphila*	Acetate, propionate	Mucin	Acetate: Acetyl-CoA pathway (acetyl-CoA synthetase); propionate: succinate pathway (methylmalonyl-CoA mutase) and propanediol pathway (propanediol dehydratase)	Provides monosaccharides to *Roseburia*; secretes succinate to *Eubacterium hallii*	Vitamin B_12_	Facchin et al. (2024), Hughes et al. (2025), Duan et al. (2023)
*Faecalibacterium prausnitzii*	Butyrate	Acetate, β-mannan oligosaccharides	Butyryl-CoA:acetate CoA-transferase pathway (HBD, BCD, BUT)	Receives β-mannan from *Bacteroide* and acetate/lactate from *Bifidobacterium*	–	Singh et al. (2022)
*Bifidobacterium* spp. (*Bifidobacterium longum*)	Acetate	Glucose, fructose, human milk oligosaccharides, polysaccharides	Bifid shunt (fructose-6-phosphate phosphoketolase)	Provides acetate and lactate to butyrate producers	–	Modesto et al. (2021)
*Eubacterium hallii*	Propionate, butyrate	Lactate, acetate, 1,2-propanediol	Propionate: propanediol pathway (PduCDE); butyrate: butyryl-CoA:acetate CoA-transferase pathway (BUT) and lactate/acetate conversion pathway	Consumes lactate, acetate and fucose from *Akkermansia muciniphila*	Vitamin B_12_	Li Q. et al. (2024), Shetty et al. (2020)
*Bacteroides* spp. (*Bacteroides thetaiotaomicron*)	Acetate, propionate	Complex polysaccharides, dietary fibre	Acetate: Acetyl-CoA pathway (acetyl-CoA synthetase); propionate: succinate pathway (methylmalonyl-CoA mutase)	Provides acetate to butyrate producers	Vitamin B_12_	Yu F. et al. (2024), Sola et al. (2026)
*Prevotella* spp. (*Prevotella copri*)	Acetate	Complex carbohydrates, dietary fibre	Acetyl-CoA pathway (acetyl-CoA synthetase)	Provides acetate to butyrate producers	–	Hays et al. (2024)
*Ruminococcus* spp. (*Ruminococcus gnavus*)	Acetate	Mucin, resistant starch	Acetyl-CoA pathway (acetyl-CoA synthetase)	Degrades mucin or starch, providing monosaccharides to *Anaerostipes*	–	Yu F. et al. (2024)
*Veillonella* spp. (*Veillonella parvula*)	Propionate	Lactate	Succinate pathway (methylmalonyl-CoA mutase)	Consumes lactate from primary fermenters	Vitamin B_12_	Yu F. et al. (2024)
*Coprococcus catus*	Propionate, butyrate	Acetate, lactate	Propionate: acrylate pathway (lactate dehydratase); butyrate: butyryl-CoA:acetate CoA-transferase pathway (BUT) and butyrate kinase pathway (butyrate kinase)	SCFAs produced can be used by other bacteria	Vitamin B_12_	Sola et al. (2026)
*Roseburia* spp.	Propionate, butyrate	Inulin, starch, glucose, fucose	Propionate: propanediol pathway (propionaldehyde dehydrogenase); butyrate: butyryl-CoA:acetate CoA-transferase pathway (BUT)	Cross-feeds with *Bifidobacterium adolescentis* and *Akkermansia muciniphila*	Propanediol pathway requires B_12_	Sola et al. (2026), Nogal et al. (2021)
*Ruminococcus obeum*	Propionate	Fucose, rhamnose	Propanediol pathway (propionaldehyde dehydrogenase)	Utilizes fucose released by *Akkermansia muciniphila*	Vitamin B_12_; folate auxotroph requires folate	Sola et al. (2026)
*Anaerostipes hadrus*	Butyrate	Lactate, acetate	Butyrate: butyryl-CoA:acetate CoA-transferase pathway (BUT)	Consumes lactate/acetate from *Bifidobacterium*; receives acetate from *Blautia*	–	Nogal et al. (2021)
*Eubacterium rectale*	Butyrate	Dietary fibre, acetate	Butyryl-CoA:acetate CoA-transferase pathway (BUT)	Receives acetate from primary fermenters	–	Hays et al. (2024)

### 2.1 Faecalibacterium prausnitzii

*Faecalibacterium prausnitzii* is an important bacterium that produces butyrate using a fixed method. It mainly employs the butyryl-CoA: acetate CoA-transferase (BUT) pathway, while the butyrate kinase pathway is not found (Singh et al., 2022). Specifically, glycolysis generates acetyl-CoA, and two acetyl-CoA molecules combine to form acetoacetyl-CoA. Then it changes to butyryl-CoA step by step under the action of 3-hydroxybutyryl-CoA dehydrogenase (HBD), dehydrogenase, and butyryl-CoA dehydrogenase (BCD). Among them, HBD catalyzes a key redox step, and its activity, along with factors such as pH, temperature, and NADH/NAD + balance, co-regulates the rate of butyrate synthesis (Shin et al., 2025; Wang Q. et al., 2026). BCD is responsible for terminal reduction and can form a complex with electron-transfer flavoproteins, maintaining the balance between oxidation and reduction within cells (Park et al., 2025). After introducing the BCD gene into a heterologous host, butyric acid production in the recombinant one increased significantly, highlighting the critical role of BCD in this process (Park et al., 2022). Finally, BUT transfers a coenzyme A group to external acetate to release butyrate, so acetate is needed for butyrate synthesis. Notably, this bacterium is weak in breaking down polysaccharides. Therefore, it needs other bacteria to help. For example, it can hydrolyze *β*-mannan released by *Bacteroides* into oligosaccharides for metabolic needs (Lindstad et al., 2021), and also utilizes human milk oligosaccharides secreted by *Bifidobacterium* (Onodera et al., 2025). This shows the significance of cooperation among various SCFA-producing bacteria. In the end, it relies on intrinsic enzymes to produce butyrate and depends on symbiotic bacteria for sufficient substrates.

### 2.2 Roseburia

*Roseburia* is one of the most abundant butyrate-producing genera in the human colon. It includes species such as *Roseburia intestinalis*, *Roseburia hominis*, and *Roseburia inulinivorans*. Its main fermentation product, butyrate, is thought to be formed via the BUT pathway (Nie et al., 2021). This route places *Roseburia* as a central acetate consumer within intestinal cross-feeding webs. For example, in co-culture with *Bifidobacterium adolescentis* on mannan-oligosaccharides, *Roseburia hominis* takes up acetate released by the latter and converts it into butyrate. Both partners reach higher cell densities than when grown alone (Bhattacharya et al., 2022). A similar interaction also occurs with the mucin degrader *Akkermansia muciniphila*. Monosaccharides derived from mucin degradation could be utilized by *Roseburia* to fuel its growth and butyrate synthesis (Pichler et al., 2020). Through these cooperative links, dietary fibers and host-derived glycans are channeled into butyrate. This may increase the overall butyrate output of the microbial community.

In addition to substrates, *Roseburia* may also depend on vitamins supplied by other gut microbes. Many *Roseburia* strains cannot synthesize thiamine or folate and thus require an external supply (Soto-Martin et al., 2020). Consequently, their abundance may be influenced by the availability of these cofactors. These may come from the diet or from neighboring bacteria. Taken together, *Roseburia* relies on exogenous acetate to produce butyrate, and its growth may further depend on vitamins provided by other community members. These intertwined dependencies position the genus as a key acetate consumer and butyrate contributor in the intestinal SCFA network.

### 2.3 Bifidobacterium

*Bifidobacterium* produces acetate and lactate primarily through the bifid shunt. This pathway uses fructose-6-phosphate phosphoketolase to split fructose-6-phosphate into acetyl phosphate and erythrose-4-phosphate. Acetyl phosphate is subsequently converted to acetate, while erythrose-4-phosphate is metabolized to lactate via glycolysis. Modesto et al. (2021) noted that the theoretical acetate-to-lactate ratio was 3:2. However, the actual ratio varies according to the substrate type, growth conditions, and other variables (Román et al., 2024). Different strains exhibit distinct metabolic capabilities and are classified into taxa such as *Bifidobacterium bifidum, Bifidobacterium animalis*, and *Bifidobacterium longum*, among others. Among these, *Bifidobacterium longum* displays the highest SCFA-producing capacity (Li W. et al., 2024). Substrate identity further shapes their fermentation profiles. Simple monosaccharides and disaccharides yield acid ratios close to the theoretical value, whereas fucosyl-lactose promotes the generation of 1,2-propanediol (1,2-PD) (Dedon et al., 2020; Tsukuda et al., 2021). Polysaccharides enhance acetate output and reduce lactate secretion, thereby supplying more substrate for butyrate-producing bacteria (Pan et al., 2022). Additionally, growth rate and nutrient availability also influence the fermentation outcome, with nutrient-poor conditions decreasing acetate and increasing lactate production (Ortiz Camargo et al., 2023).

The acetate released by *Bifidobacterium* serves as a key substrate for cross-feeding and can be directly converted to butyrate by butyrate-producing bacteria. In turn, sugars liberated by these bacteria can be reutilized by *Bifidobacterium*, forming a mutualistic metabolic cycle (Chia et al., 2021). This relationship is context-dependent, however; when competing for the same substrates, butyrate production may decline (Xiao et al., 2024). In summary, *Bifidobacterium* generates acetate and lactate under the combined influence of strain type, substrate availability, and growth state. Through acetate-mediated cross-feeding, it actively participates in carbon flow distribution within SCFA-producing networks.

### 2.4 Eubacterium hallii

*Eubacterium hallii* is also a typical SCFA-producing bacterium. It cannot break down complex sugars, but it can use metabolites from other bacteria and plays a vital role in the formation of SCFAs (Gu et al., 2025). It mainly produces butyrate and propionate. There are two methods to produce butyrate. On the one hand, it produces this metabolite via the BUT pathway; on the other hand, it converts lactic acid and acetate from other bacteria into butyrate (Shetty et al., 2020), giving it a competitive advantage in lactic acid-rich environments. The efficiency of this process depends on the substrate concentration, and the relative ratio of lactic acid to acetic acid determines butyrate yield (Mukhopadhya and Louis, 2025).

It is also a source of propionate. It employs cobalamin-dependent glycerol/diol dehydratase (PduCDE) to transform 1,2-PD into propionate (Li Q. et al., 2024). In infants, *Bifidobacterium* breaks down human milk oligosaccharides to release fucose, lactic acid, and acetic acid. At this time, *Eubacterium hallii* converts them into butyrate and propionate, thereby promoting the maturation of the infant’s SCFA pool (Pham et al., 2022; Wang X. et al., 2026). Metagenomic data show that the PduCDE gene of this bacterium was detected in 63–81% of adult gut samples. This indicates its wide existence in the human intestine (Ramirez Garcia et al., 2023). Besides, it can produce vitamin B_12_, fulfilling its metabolic needs. In parallel, this vitamin may also be used by other B_12_-requiring bacteria, potentially stabilizing the network under nutrient-scarce conditions (Seegers et al., 2021). To summarize, this bacterium produces butyrate and propionate by utilizing lactic acid and 1,2-PD. Its self-synthesis of vitamin B_12_ makes it a vital component in the intestinal SCFA-producing network.

### 2.5 Other SCFA-producing gut bacteria

**Figure 2:**
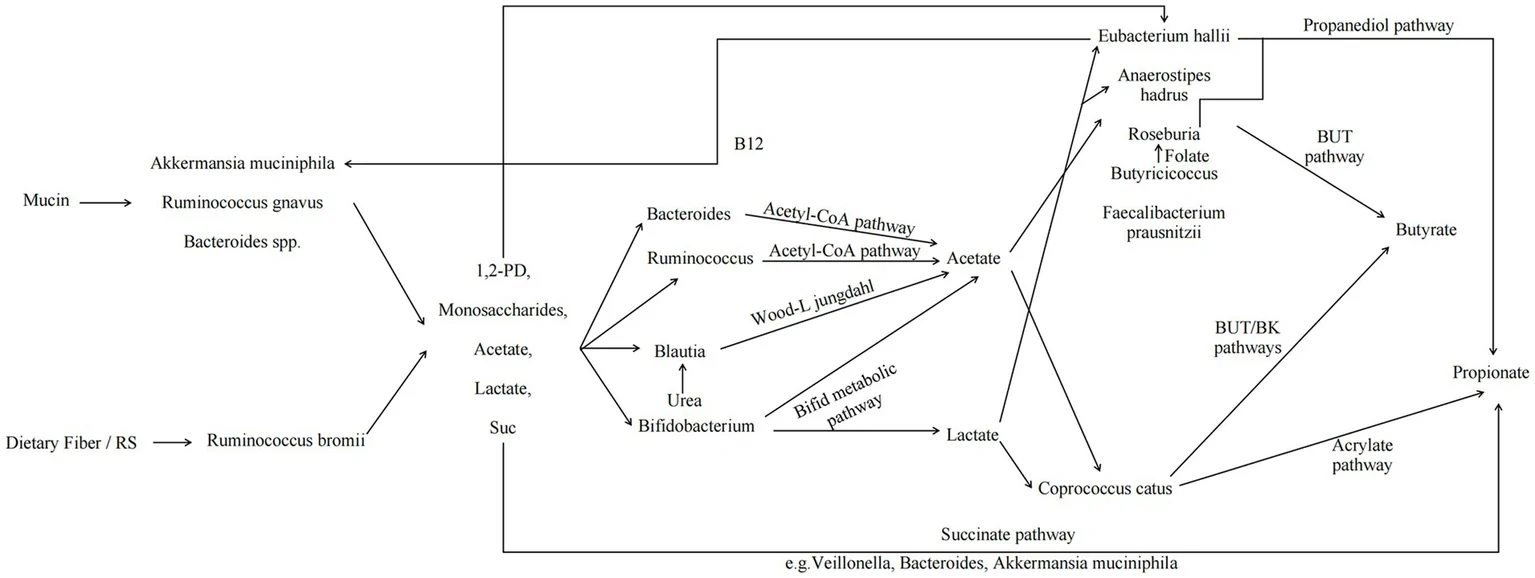
Cross-feeding among short-chain fatty acid-producing bacteria: Primary degraders such as *Akkermansia*, *Ruminococcus*, and other genera break down host-derived mucin, dietary fiber, and resistant starch (RS) into monosaccharides, 1,2-propanediol (1,2-PD), succinate (Suc), lactate, acetate, and other intermediates. These metabolites are subsequently utilized by *Bifidobacterium*, *Blautia*, and other secondary fermenters via distinct metabolic pathways to yield acetate, propionate, and butyrate. In addition, certain taxa, including *Eubacterium hallii* and *Butyricicoccus*, are capable of releasing vitamin B_12_ and folate, which serve as essential cofactors for the metabolism of neighboring bacteria.

Beyond the genera discussed above, the intestinal SCFA network encompasses a broader range of taxa that contribute to acetate, propionate, and butyrate pools through metabolic complementarity and cross-feeding. Several of these genera, including *Akkermansia muciniphila, Ruminococcus, Anaerostipes, Butyricicoccus, Blautia,* and *Coprococcus*, play distinct roles: the former two degrade mucin and carbohydrates, providing metabolic substrates and SCFAs, whereas the latter four mainly convert these substrates into SCFAs. Figure 2 illustrates a schematic diagram of cross-feeding among different SCFA-producing bacteria.

*Akkermansia muciniphila* resides in the intestinal mucus layer. Its main function is to degrade mucin, providing metabolic substrates for other bacteria, while also producing small amounts of acetate and propionate (Panzetta and Valdivia, 2024). Functionally, it divides mucin into fucose, N-acetylglucosamine, and other simple sugars (Aja et al., 2025). These sugars are converted into pyruvate (Pyr) via glycolysis (Lee et al., 2022), the essential precursor for both acetate and propionate. For acetate, Pyr is converted to acetyl-CoA and then to acetate, supplying energy to itself and other bacteria (Facchin et al., 2024). For propionate, a portion of pyruvate is carboxylated to succinate (Suc). In *Akkermansia muciniphila*, the conversion of Suc to propionate requires vitamin B_12_ (Hughes et al., 2025). Under B_12_-limiting conditions, *Akkermansia muciniphila* secretes Suc, which is utilized by propionate producers, such as *Eubacterium hallii*. Importantly, only one-third of such bacteria can produce vitamin B_12_ by themselves. Therefore, most bacteria need to get it from the environment, leading to strain-specific SCFA profiles (Kirmiz et al., 2020; Aja et al., 2025). Additionally, fucose metabolism yields 1,2-PD, which is rapidly converted to propionate by other community members, further contributing to propionate production (Høgsgaard et al., 2023).

*Ruminococcus* is among the genera that produce acetate via the acetyl-CoA pathway, with *Ruminococcus gnavus* and *Ruminococcus bromii* as representative species (Hong et al., 2024). *Ruminococcus gnavus* acts as a mucin degrader, encoding sialidases, fucosidases, and other glycoside hydrolases. Together with *Akkermansia muciniphila*, *Bacteroides* species, and *Ruminococcus torques*, *Ruminococcus gnavus* is responsible for the bulk of mucin O-glycan depolymerization in synthetic communities (Berkhout et al., 2024). The monosaccharides released can be taken up by butyrate producers such as *Anaerostipes caccae*, thereby indirectly supporting butyrate formation. *Ruminococcus bromii* functions as a keystone degrader of resistant starch, liberating glucose and malto-oligosaccharides that serve as substrates for *Ruminococcus gnavus* and other bacteria (Van-Wehle and Vital, 2024). The acetate output of this genus therefore depends on both its own metabolic routes and the substrate supply from primary degraders within the community.

*Anaerostipes* is an important butyrate-producing genus. This genus generates butyrate via two main routes. The first one is the BUT pathway. The second metabolic pathway links lactate with acetate. A lactate dehydrogenase–electron-transfer flavoprotein complex oxidizes lactate into pyruvate; the generated pyruvate then flows into the acetyl-CoA pathway to synthesize butyrate (Shetty et al., 2020). This latter pathway confers a distinct advantage in lactate-rich intestinal environments (Liu et al., 2024). Under SCFA-replete conditions, *Anaerostipes hadrus* can also employ the reverse *β*-oxidation pathway. It condenses exogenous acetate and propionate into valerate and hexanoate (Fitzgerald et al., 2025). This suggests that its fermentation profile is not fixed; shifts in substrate type directly influence the chain length of the final products. In addition, carbohydrate utilization capacity varies considerably among strains, further shaping the overall SCFA composition (Herold et al., 2025).

*Butyricicoccus*, a member of *Clostridium* cluster IV, synthesizes butyrate predominantly through the BUT pathway. Beyond this canonical function, genomic analyses have revealed that *Butyricicoccus porcorum* harbors genes implicated in folate biosynthesis and one-carbon metabolism (Zhao et al., 2025). Folate is an important metabolite in microbial cross-feeding. Some butyrate producers, such as certain *Roseburia* strains, are folate auxotrophs and may rely on other community members for folate supply (Soto-Martin et al., 2020). The folate synthesis capacity of *Butyricicoccus porcorum* could therefore indirectly support the butyrate-producing network. A further potential advantage comes from its one-carbon metabolism, which provides key precursors for nucleotide synthesis and methylation reactions, enhancing its competitiveness (Ota and Takahashi, 2026). Additionally, the abundance of *Butyricicoccus* may be influenced by dietary factors, as high intake of barley and natto has been associated with increased levels of this genus in healthy individuals (Maruyama et al., 2024). These metabolic features offer new clues to its ecological role, but their functional significance remains to be confirmed.

*Blautia* is an anaerobic genus within the *Lachnospiraceae* family. Its main fermentation product is acetate. Unlike most acetate producers that rely on sugar fermentation, some strains can synthesize acetate directly from H_2_ and CO_2_ via the Wood–Ljungdahl pathway. This unique metabolic feature is defined as acetogenesis (Firth et al., 2025). It allows acetate production to continue even when sugars are scarce. Certain species, such as *Blautia coccoides*, can also produce propionate and butyrate (Holmberg et al., 2024). This acidogenic capacity is closely tied to urease activity. Urease-encoding strains hydrolyze urea into ammonia and CO_2_. The released CO_2_ is recycled into acetate through the Wood–Ljungdahl pathway, directly incorporating urea carbon into the SCFA pool (Firth et al., 2025). This route does not depend on dietary carbohydrates. In co-culture with *Anaerostipes hadrus*, this acetate is converted to butyrate, further channeling urea carbon into butyrate. Additionally, *Blautia* can extend exogenous acetate and propionate to valerate through reverse *β*-oxidation, thereby enriching the species and contents of intestinal SCFAs (Fitzgerald et al., 2025).

*Coprococcus* is a butyrate-producing genus, including *Coprococcus eutactus* and *Coprococcus catus*, among others (Notting et al., 2023). Its main fermentation product is butyrate, synthesized via the BUT pathway. The two species exhibit distinct acidogenic features. *Coprococcus eutactus* is one of the few gut bacteria possessing two terminal enzymes for butyrate synthesis, BUT and butyrate kinase, thus harboring dual routes for butyrate formation (Notting et al., 2023). *Coprococcus catus*, by contrast, can additionally utilize lactate to generate propionate through the acrylate pathway and convert part of the acetate into extra butyrate (Sheridan et al., 2022). In this species, the acrylate pathway genes are strongly upregulated by lactate, and this induction is not suppressed by fructose. This contrasts with certain other lactate-utilizing bacteria. Consequently, it maintains lactate conversion into propionate and butyrate even in sugar-rich environments. And the resulting SCFAs can be fed into cross-feeding networks for use by other butyrate producers.

## 3 The association between SCFA-producing bacteria and gallstones

The former section has shown the metabolic features of typical SCFA-producing bacteria, clarifying how these bacteria form a highly efficient metabolic network through cross-feeding. Beyond SCFA production, these bacteria have also drawn attention for their possible involvement in gallstone pathogenesis. A growing number of observational, genetic, and animal studies have reported associations between SCFA-producing bacteria and reduced gallstone risk. Thus, this section summarizes these findings, tracing the evidence from correlative links to suggestive causal relationships and functional corroboration in animal experiments.

### 3.1 Observational research

Observational research provides a foundation for identifying associations. Table 3 presents the observational study results from subjects with gallstones. By comparing the structure of intestinal and biliary flora in gallstone and healthy subjects, many studies have found that SCFA-producing bacteria are substantially less abundant under pathological states. However, dietary habits, age, and medication use may confound such comparisons, thereby weakening the accuracy of the results. In fecal samples, Chen et al. (2021) found that the quantity of *Lachnospiraceae*_UCG-008, *Lachnospiraceae*_UCG-010, as well as *Prevotellaceae*_UCG-001 was markedly reduced in gallstone model mice; Georgescu et al. (2022) further extended the subjects from animals to middle-aged and elderly patients, and found the abundances of *Faecalibacterium prausnitzii* and *Dorea* spp. were lower; Chang et al. (2024) likewise noticed decreased *Prevotellaceae_*2 and *Ruminococcus_*2, with diminished fecal butyrate levels among Taiwanese patients. This finding links gut microbiota structure to metabolism, suggesting these bacteria may be associated with gallstone formation potentially via their metabolites.

**Table 3:** Comparison of gut and biliary microbiota in gallstone subjects.

Gut/biliary microbiota	Decreased taxa	Increased taxa	Subjects	Sample type	References
Gut microbiota	—	*Desulfovibrionales*	Cholesterol gallstone patients	Feces	Hu et al. (2022)
	*Faecalibacterium* *Prevotella*_9 *Bacteroides plebeius* DSM 17135	*Bacteroides uniformis*	Cholesterol gallstone patients	Feces	Chang et al. (2024)
	*Akkermansia* *Prevotella* *Bifidobacterium* *Alistipes* *Bacteroides* *Dorea* *Ruminococcus* *Faecalibacterium* *Methanobrevibacter* *Faecalibacterium prausnitzii* *Bifidobacterium adolescentis* *Methanobrevibacter smithii*	—	Cholesterol gallstone patients	Feces	Georgescu et al. (2022)
	*Faecalibacterium prausnitzii Lactobacillus rogosae hermodesulfovibrio thiophilus*	*Clostridium glycyrrhizinilyticum* *Clostridium perfringens*	C57BL/6J mice	Feces	Liu et al. (2025a)
	*Akkermansia muciniphila* CAG-448	*Bacteroides stercorirosoris* *Enterocloster*	Cholesterol gallstone mice	Feces	Bai H. et al. (2025)
Biliary microbiota	—	*Alcaligenaceae*	Primary and secondary bile duct stone patients	Bile	Feng et al. (2022)
	—	*Cutibacterium acnes* *Microbacterium sp005774735* *Agrobacterium pusense* *Enterovirga sp013044135*	Cholesterol gallstone patients	Bile and gallstones	Zhang et al. (2024)
	*Actinomyces* *Proteus* *Clostridium_sensu_stricto* *Klebsiella* *Actinobacillu* *Lachnospiraceae*_UCG-008 *Butyrivibrio* *Roseburia* *Porphyromonas* *Streptococcus* *Helicobacter* *Enterobacter*	*Brevundimonas and Prevotella_*1	Primary bile duct stone patients	Bile	Lyu et al. (2021)
	*Bacteroidetes* and *Actinobacteria*	*Synergistetes*	Recurrent and primary bile duct stone patients	Bile	Tan et al. (2022)

**Figure 3:**
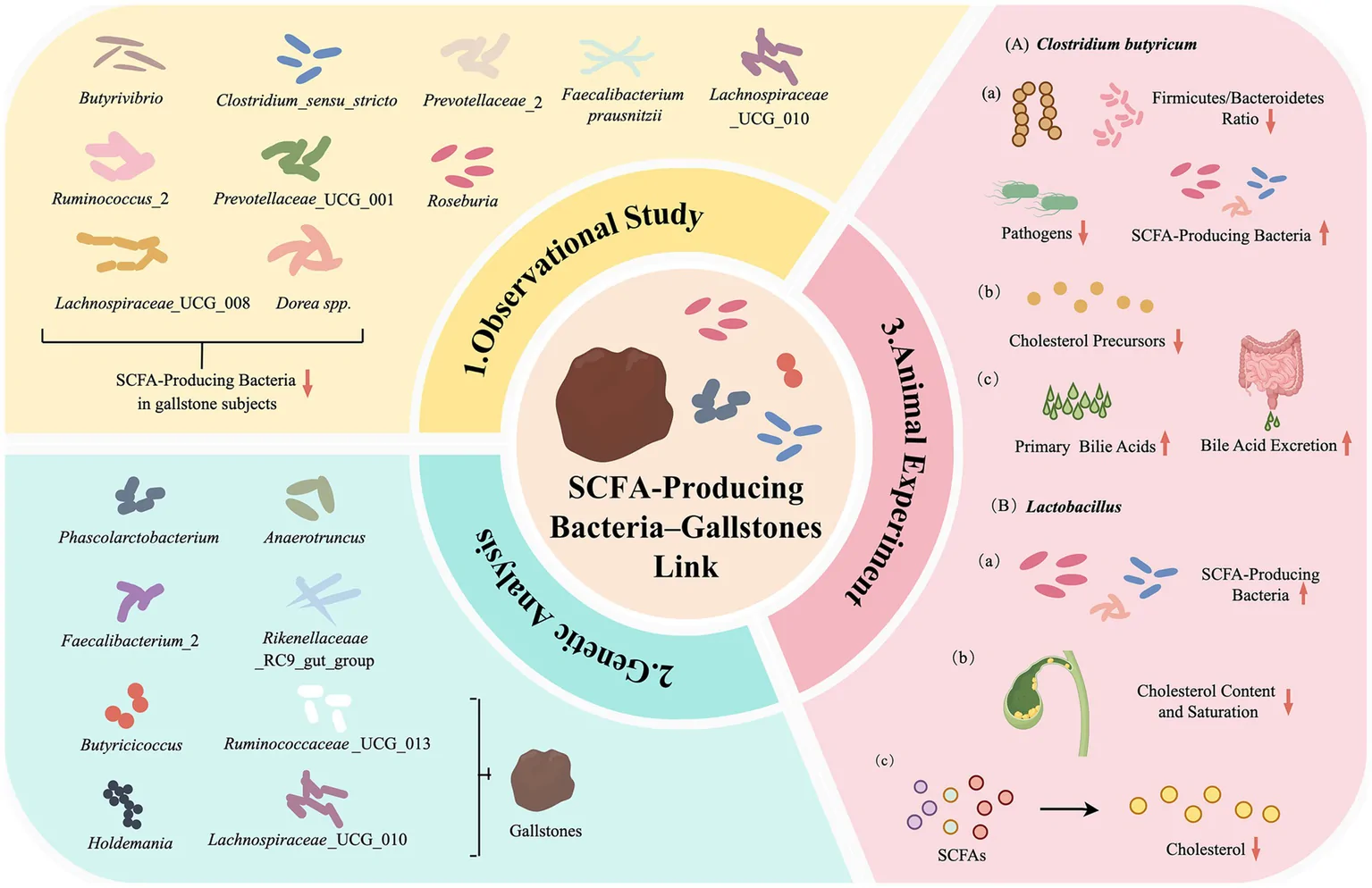
Evidence linking short-chain fatty acid (SCFA)-producing bacteria to gallstones from three perspectives: 1. Observational studies: The abundance of multiple SCFA-producing bacteria (such as *Butyrivibrio*, *Faecalibacterium prausnitzii*, *Roseburia*) has been found to be reduced in gallstone subjects, suggesting a potential link between these bacteria and gallstone disease. 2. Genetic analysis: Genetic features of several SCFA-producing genera (such as *Phascolarctobacterium*, *Butyricicoccus*, *Holdemania*) have been associated with gallstone risk, providing suggestive evidence for a possible causal relationship. 3. Animal experiments: Anti-lithogenic mechanisms of representative SCFA-producing strains have been explored in animal models: **(A)** *Clostridium butyricum* appears to lower the Firmicutes/Bacteroidetes ratio and pathogen abundance, while potentially enriching SCFA-producing bacteria. It may also decrease cholesterol precursors, promote primary bile acid synthesis, and enhance bile acid excretion; **(B)** *Lactobacillus* strains have been reported to elevate the abundance of SCFA-producing bacteria. They may reduce cholesterol content and saturation in bile, and could directly lower cholesterol levels via SCFA metabolites.

Further studies focused on the biliary microenvironment, which explores the relationship between bile flora and gallstones. Lyu et al. (2021) analyzed bile microbiota profiles in patients with gallstones and found that the amount of *Clostridium*, *Lachnospiraceae* UCG_008, *Butyrivibrio*, and *Roseburia* was substantially lower than that in controls. This study extended the correlation analysis from the gut to the biliary tract. Compared with fecal samples that only reflect intestinal flora, bile samples from lesion sites may more directly reveal stone-related microbial alterations, offering more targeted correlational evidence. Collectively, despite differences in subjects, regions, and detection methods, the consistently observed reduction of SCFA-producing bacteria in the gut and biliary tract of these subjects points to a potential link with gallstones. Figure 3, panel 1 shows the SCFA-producing bacteria that were observed to be decreased in subjects with gallstones.

### 3.2 Genetic study

Observational research cannot confirm causality. Mendelian randomization, which uses genetic variants to reduce confounding and reverse causation, could offer suggestive evidence relevant to causal inference. Li W. et al. (2023) utilized large-scale genomic data to reveal that *Phascolarctobacterium*, *Rikenellaceae* RC9_gut_group, and *Ruminococcaceae* UCG_013 are associated with a decreased gallstone risk. It supports beneficial roles for these bacteria.

Additional genetic analyses have identified further protective taxa. Liu X. et al. (2023) reported that elevated *Holdemania* and *Lachnospiraceae* UCG_010 reduced gallstone risk. The protective effect of *Butyricoccus* has also been suggested (Yin and Zhang, 2024). More importantly, these studies also reveal functional heterogeneity among strains of the same taxon. Liu et al. noted that *Faecalibacterium*_2 reduced gallstone risk, while *Faecalibacterium*_3 increased it, possibly due to their different bile salt hydrolase (BSH) activity (Liu X. et al., 2023; Zhao J. et al., 2024). BSH is an enzyme involved in the regulation of bile acids. This finding points to abnormal bile acid metabolism as a potential key contributor to gallstones. It also suggests that common SCFA-producing capacity may not fully account for strain-specific influences on gallstones. Strain-specific metabolic features may be more critical than SCFA yields. Moreover, these insights offer preliminary implications for future clinical treatment and daily health regulation. If confirmed by further mechanistic studies, the development of targeted probiotic products should extend beyond genus classification and focus on specific strains. A comprehensive assessment of metabolic properties, including BSH activity, would be essential. Figure 3, panel 2 shows the SCFA-producing bacteria that were found to reduce the risk of gallstones in Mendelian randomization.

### 3.3 Animal experiments

Animal tests offer experimental support for their stone-inhibiting effects. Most of the experiments focus on *Clostridium butyricum*. In 1983, Takeda et al. conducted animal studies and were the first to report that a single SCFA-producing bacterial strain exerts an anti-lithogenic effect in animal models (Takeda et al., 1983). In mice with cholesterol gallstones, feeding *Clostridium butyricum* was found to lower gallstone levels and reduce bile cholesterol. Long-term use even appeared to dissolve formed gallstones. This study provided initial animal evidence that such bacteria may have the potential to prevent and treat gallstones. On this basis, Li et al. further explored its regulatory mechanism. This bacterium was shown to adjust intestinal flora, such as lowering the ratio of Firmicutes to Bacteroidetes, decreasing harmful bacteria, and increasing SCFA-producing bacteria. It also reduces cholesterol precursors, raises primary bile acids, and speeds up bile acid excretion in murine models, suggesting a protective effect (Li G. et al., 2023).

Besides *Clostridium butyricum,* some *Lactobacillus* strains have shown good stone-preventing ability in animal studies. Although they mainly produce lactic acid, heterofermentative strains such as *Lactobacillus reuteri* and *Lactobacillus plantarum* can produce acetic acid, recognized as minor contributors to the SCFA pool. They have been observed to increase SCFA-producing bacteria and help keep intestinal flora stable. In parallel, treatment with *Lactobacillus* reduced the concentrations of tauro-*α*-muricholic acid and tauro-*β*-muricholic acid. This relieved their inhibition of farnesoid X receptor (FXR). Following its activation, ileal fibroblast growth factor 15 (FGF15) was upregulated. FGF15 then reached the liver and bound to hepatic fibroblast growth factor receptor 4. Subsequently, this binding led to elevated expression of small heterodimer partner. In turn, SHP suppressed cholesterol 7α hydroxylase and oxysterol 7α hydroxylase, two key enzymes in bile acid synthesis. Meanwhile, *Lactobacillus* also upregulated hepatic transporters. These include multidrug resistance-associated protein homologs 3 and 4, multidrug resistance protein 2, and the bile salt export pump. In doing so, it enhanced bile acid excretion. Through the combined regulation of bile acid synthesis and excretion, biliary cholesterol saturation was ultimately reduced. Moreover, they may modulate lipid metabolism by the SCFAs they secrete, potentially inhibiting cholesterol gallstone formation through multiple mechanisms (Ye et al., 2022; Che et al., 2025; Wang et al., 2025). Figure 3, panel 3 shows the potential gallstone-protective bacteria and mechanisms identified in animal experiments.

At the same time, it should be noted that beneficial effects cannot be assumed to be generalizable across all SCFA-producing bacteria. This is highlighted by the divergent associations of different *Faecalibacterium* species with gallstone risk. Strain-level functional properties, such as BSH activity, substrate utilization profiles, and cross-feeding capabilities, are likely critical determinants of anti-lithogenic efficacy. Moreover, preclinical research mostly adopts rodent gallstone models constructed with cholesterol- and cholic acid-enriched lithogenic diets. Such models fail to completely mimic the multifactorial pathogenesis of human cholelithiasis. In addition, disparities between species in gut anatomy and bile acid profiles may interfere with the reliability of translational inference. These considerations underscore the need for human-relevant model systems and strain-level characterization in future investigations. In short, SCFA-producing bacteria, including *Clostridium butyricum* and *Lactobacillus*, have shown promise in potentially counteracting gallstone formation via multiple pathways in animal experiments. This expands the range of candidate bacteria for gallstone intervention and may offer a foundation for future exploration of non-surgical treatment options.

## 4 Anti-gallstone mechanisms of gut microbiota-derived SCFAs

A growing body of research has recognized an association between SCFA-producing bacteria and gallstone prevention. This may be related to their metabolites. Among the various metabolites produced by these bacteria, SCFAs are considered to have potential protective effects against gallstones. However, it should be noted that SCFAs may not be the sole mediating factor. Gallstones are mainly classified into cholesterol stones and pigment stones, with the latter further subdivided into black and brown subtypes. Cholesterol stones are primarily associated with biliary cholesterol supersaturation, disordered bile acid metabolism, mucin hypersecretion, and other factors (Grigor’eva and Romanova, 2020). Pigment stones are primarily composed of calcium bilirubinate and other substances. Black pigment stones are commonly found in patients with hemolysis, cirrhosis, or cystic fibrosis. These pathological conditions can increase the production of calcium bilirubinate. Brown pigment stones are closely associated with biliary infection, and their formation depends on the functions of bacterial enzymes such as *β*-glucuronidase and phospholipase (Costa et al., 2024). Notably, since most evidence concerns cholesterol stones, the mechanisms below are in the context of this one; their relevance to pigment stones is assessed in each section, as shown in Table 4. Based on this, this part mainly analyzes the anti-gallstone mechanisms of SCFAs from five aspects, including regulating lipid metabolism, modulating bile acid metabolism, maintaining the intestinal barrier, balancing gut flora, and relieving cholecystitis.

**Table 4:** Summary of SCFA anti-gallstone mechanisms and their relevance to different stone types.

Anti-gallstone mechanism	Evidence for cholesterol stones	Evidence for pigment stones
Lipid metabolism regulation	Correlation supported (regulates synthesis, absorption, excretion; reduces cholesterol saturation)	No correlation supported
Bile acid metabolism modulation	Correlation supported (differential FXR regulation, BSH-promoted excretion, lowers hydrophobicity index)	No correlation supported
Intestinal barrier maintenance	Correlation supported (prevents LPS translocation, reduces MUC5AC and cholesterol nucleation)	Correlation supported for brown pigment stones (prevents translocation of enzyme-producing bacteria, inhibits calcium bilirubinate and calcium soap)
Gut microbiota optimization	Correlation supported (corrects dysbiosis, reduces pro-inflammatory and pro-lithogenic factors)	Correlation supported for brown pigment stones (suppresses enzyme-producing pathogens, attenuates enzyme activity via acidification)
Cholecystitis alleviation	Correlation supported (reduces TNF-α and other inflammatory cytokines, attenuates MUC5AC stimulation)	Correlation supported for pigment stones (preserves MRP2 function, inhibits endogenous β-glucuronidase, maintains contractility to reduce calcium deposition)

### 4.1 Lipid metabolism regulation

**Figure 4:**
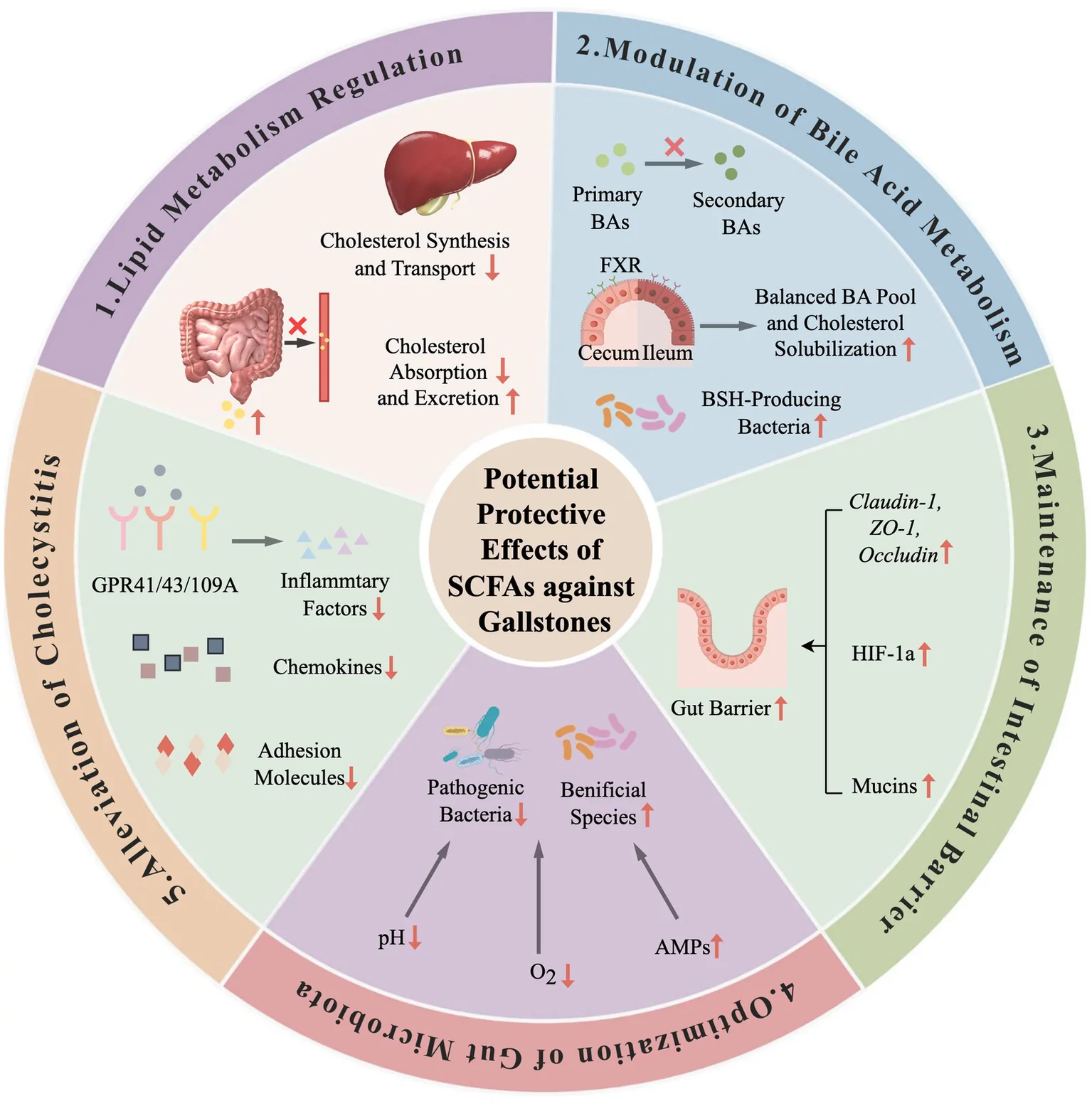
Potential mechanisms of short-chain fatty acids (SCFAs) in gallstone prevention: Lipid metabolism regulation: SCFAs may suppress hepatic cholesterol synthesis and transport, while potentially reducing intestinal absorption and promoting fecal excretion, thereby helping to lower biliary cholesterol saturation.2. Modulation of bile acid metabolism: SCFAs appear to regionally modulate FXR signaling, may increase bile salt hydrolase (BSH)-producing bacteria, and could lower intestinal pH to limit secondary bile acid conversion, which might optimize the bile acid pool and reduce cholesterol precipitation.3. Maintenance of intestinal barrier: SCFAs are thought to upregulate tight junction proteins (claudin-1, ZO-1, occludin), possibly stimulate mucin secretion, and may help stabilize HIF-1*α*, thereby potentially reinforcing gut barrier integrity and limiting bacterial and LPS translocation.4. Optimization of gut microbiota: SCFAs may acidify the intestinal lumen, induce physiological hypoxia, and promote antimicrobial peptide (AMP) secretion, which could suppress pathogens and enrich beneficial taxa. 5. Alleviation of cholecystitis: SCFAs may reduce inflammatory factors via GPR receptors and could also decrease chemokines and adhesion molecules, potentially relieving cholecystitis.

Cholesterol supersaturation is the primary cause of cholesterol gallstones (Portincasa et al., 2023). By regulating the synthesis, absorption, and discharge of cholesterol, SCFAs could help reduce their levels and curb gallstone development, as shown in Figure 4, panel 1. Butyric acid appears to act as an inhibitor of cholesterol synthesis. It seems to suppress 3-hydroxy-3-methylglutaryl coenzyme A reductase activity. This would slow cholesterol production. It also appears to downregulate H3K27 and H3K9 histone acetylation. This epigenetic change could reduce expression of synthesis-related genes such as HMGCR and squalene monooxygenase (Zhong et al., 2025). Propionate might also restrict liver cholesterol synthesis via immune-dependent pathways. This mechanism differs from that of butyric acid (Haghikia et al., 2021). In addition, acetic acid combined with propionate has been reported to produce a stronger lipid-lowering effect. This finding suggests that various SCFAs could work together to boost efficacy (Chang et al., 2026). Therefore, raising multiple SCFAs via diet might bring better anti-gallstone effects than taking only one type.

In the absorption and discharge of cholesterol, butyrate appears to serve both functions. It has been shown to reduce cholesterol absorption in the intestine by downregulating Niemann-Pick C1-Like 1. It also elevates ATP-binding cassette subfamily G member 5/8 transporters (ABCG5/8) (Zhong et al., 2025). This would boost cholesterol excretion via feces. Propionate exhibits similar effects on the absorption of cholesterol (Chen and Ho, 2023).

The role of acetate in cholesterol metabolism seems contradictory. On one hand, it can be used to produce cholesterol (Borcik et al., 2022). On the other hand, it can also be converted into butyrate to lower cholesterol through cross-feeding. The net effect of acetate is likely to depend on the prevailing metabolic context. Under conditions favoring cross-feeding, acetate tends to be channeled toward butyrate production. This would result in an overall cholesterol-lowering effect. Such conditions include high fiber intake and an intact network of butyrate-producing bacteria. Conversely, when cross-feeding is impaired due to dysbiosis, insufficient substrate supply, or disrupted microbial networks, acetate could instead be diverted toward hepatic cholesterol synthesis. This context dependence implies that acetate supplementation alone is unlikely to yield predictable anti-lithogenic benefits. The presence of acetate-utilizing butyrate producers would probably be necessary for consistent protection.

Moreover, SCFAs appear to inhibit lecithin-cholesterol acyltransferase activity. This would reduce plasma cholesterol transport and decrease its content in bile (Zhou et al., 2023). This effect is indirect. It extends the lipid-lowering regulation from locality to systematization. Since pigment stones form via calcium bilirubinate precipitation rather than cholesterol supersaturation, the cholesterol-lowering effects of SCFAs are unlikely to directly prevent pure pigment stones. Indirect benefits might exist in mixed stones or when accompanied by cholesterol metabolism disorders. In a nutshell, SCFAs jointly regulate cholesterol synthesis, absorption, excretion, and transport. This could help lower bile cholesterol saturation. It might ultimately contribute to the prevention of cholesterol gallstone formation.

### 4.2 Modulation of bile acid metabolism

SCFAs might exert protective effects against gallstones by modulating bile acid metabolism. One proposed mechanism involves FXR signaling along the intestinal tract (Li et al., 2026). It has been reported that in the cecum, SCFAs appear to activate FXR (Ye et al., 2021). This would promote cholesterol solubilization. In the ileum, they seem to suppress FXR (Xu et al., 2022). This regional divergence could reflect differences in local SCFA levels, microbial composition, and baseline receptor expression. Functionally, such dual modulation might help maintain a balanced bile acid pool and facilitate cholesterol dissolution. However, SCFAs regulate FXR indirectly through its ligands and antagonists. The net effect is likely shaped by the microbiota, bile acid profile, diet, and circadian rhythms. Whether these short-term adjustments translate into lasting protection remains uncertain.

Another related route involves microbial bile acid-metabolizing enzymes. SCFAs have been reported to increase the abundance of BSH-producing bacteria, including certain *Lactobacillus* species (Ye et al., 2021). BSH catalyzes the deconjugation of conjugated bile acids (Guzior and Quinn, 2021). This deconjugation has been proposed to reduce bile acid reabsorption and promote their excretion. In turn, this could help lower biliary cholesterol saturation (Yang et al., 2021). However, the relationship between BSH activity and gallstone formation appears not straightforward. Elevated BSH has been observed in gallstone patients, independent of total bacterial load (Wang et al., 2020). This apparent discrepancy might reflect a compensatory response to a more hydrophobic bile acid pool rather than a primary protective driver. It could also be due to functional differences among BSH isoforms with distinct substrate specificities. Alternatively, BSH enrichment under dysbiosis might not confer the same benefits as in a healthy microbiota. Resolving this will require mechanistic studies that distinguish BSH isoforms and define their specific contributions to bile acid homeostasis in gallstone-relevant models.

Additionally, SCFAs appear to suppress the conversion of primary bile acids into secondary bile acids. Since secondary species are more hydrophobic, this conversion raises the hydrophobicity index of the bile acid pool (Cai et al., 2022). A higher index tends to encourage cholesterol precipitation and microcrystal formation. *In vitro* data suggest that SCFAs may lower intestinal pH. This pH reduction could limit this bacterial transformation (Danladi et al., 2022). However, the specific bacterial targets, such as 7α-dehydroxylating species, have not been fully defined.

Conjugated bilirubin is highly water-soluble in bile. This keeps it stably dissolved in the biliary microenvironment and prevents precipitation under normal conditions. However, in pathological states such as infection and cirrhosis, conjugated bilirubin can be hydrolyzed. This reaction yields poorly soluble unconjugated bilirubin. Unconjugated bilirubin readily binds calcium ions, forming calcium bilirubinate (Balu et al., 2026). However, it remains unknown whether SCFAs can block such precipitation by regulating bile acid metabolism, and relevant direct evidence is still lacking. Despite these limitations, SCFAs could offer protection against cholesterol gallstones by regulating multiple steps of bile acid metabolism, remodeling the bile acid pool composition, and alleviating biliary cholesterol supersaturation. Figure 4, panel 2 illustrates these effects of SCFAs.

### 4.3 Maintenance of intestinal barrier

The disrupted intestinal barrier is thought to contribute to gallstone development (Komorniak et al., 2024). When the intestinal wall is damaged, harmful bacteria and lipopolysaccharide (LPS) can translocate into the bile tract via the portal vein or lymphoid tissue (Meacci et al., 2025). LPS appears to stimulate gallbladder epithelial cells to secrete mucin (Zhao Z. et al., 2024). This would promote cholesterol crystal nucleation and accelerate stone growth. Simultaneously, LPS could activate the MyD88-NF-κB pathway. This activation might drive continuous secretion of mucin 5 AC (MUC5AC) and mucin 2 (MUC2). It could further facilitate cholesterol precipitation. SCFAs might block this pathogenic cascade through multiple barrier-strengthening mechanisms.

SCFAs could reinforce the intestinal barrier in three main ways. The first is the mechanical barrier. Butyrate has been shown to upregulate tight junction proteins, including *claudin-1, zonula occludens-1(ZO-1),* and *occludin*. This occurs via Signal Transducer and Activator of Transcription 3 and Specificity Protein 1 pathways (Hays et al., 2024). It also seems to enhance their expression and assembly through Adenosine Monophosphate-activated Protein Kinase and Protein Kinase B pathways (Xu et al., 2023). Acetate and propionate can similarly increase *claudin-4* and *ZO-1* levels (Saleri et al., 2022).

The second is the chemical barrier. Butyrate appears to activate the MUC2 promoter. It stimulates goblet cells to secrete MUC2. This thickens the intestinal mucus layer (Liang et al., 2022). Although intestinal MUC2 and biliary MUC5AC are both mucins, they exert opposing effects on gallstone formation due to their distinct locations. MUC2 reinforces the gut barrier to prevent bacterial translocation. Biliary MUC5AC promotes cholesterol nucleation.

The third involves stabilization of Hypoxia-inducible Factor-1α.(HIF-1α) As the primary energy source for colonocytes, butyrate consumes intestinal oxygen through *β*-oxidation. This metabolic activity could help maintain HIF-1α stability (Ornelas et al., 2023). It also appears to block HIF-1α degradation by inhibiting prolyl hydroxylase (Wang et al., 2021). Stable HIF-1α might further promote mucin and tight junction protein production. This would strengthen barrier integrity.

In the context of pigment gallstones, SCFAs could exert protective effects through multiple cooperative mechanisms. *β*-Glucuronidase is secreted by bacteria that translocate from the gut. This enzyme hydrolyzes conjugated bilirubin into unconjugated bilirubin, then forms calcium bilirubinate. Phospholipase hydrolyzes biliary lecithin. This reaction generates free fatty acids and calcium soaps. All these products are constituents of brown pigment stones. SCFAs seem to strengthen the intestinal barrier. This would limit the translocation of the enzyme-producing bacteria and thereby help inhibit the formation of brown pigment stones. Additionally, SCFAs appear to reduce gallbladder mucin secretion. Such suppression helps block the development of this type of stone (Dan et al., 2023). In summary, SCFAs could enhance the intestinal barrier through multiple mechanisms, potentially limiting the translocation of bacteria and LPS, helping reduce the production of mucin and bacterial enzymes. These effects may slow the formation of cholesterol and brown pigment stones. Figure 4, panel 3 illustrates this process.

### 4.4 Optimization of gut microbiota

Gut dysbiosis is linked to gallstone formation. It is characterized by an expansion of pathogenic bacteria and a reduction in beneficial species (Grigor’eva and Romanova, 2020). This imbalance disrupts bile acid metabolism. It also promotes the release of pro-inflammatory factors that facilitate cholesterol precipitation. By lowering intestinal pH, inducing physiological hypoxia, and boosting antimicrobial peptide secretion (AMP), SCFAs could correct this dysbiosis and potentially slow gallstone progression.

In terms of pH regulation, SCFAs release hydrogen ions in the colon. This helps maintain an acidic environment. When pH drops from 6.5 to 5.5, beneficial bacteria such as *Lachnospiraceae* and *Lactobacillus* tend to increase markedly, while pathogenic bacteria like *Escherichia coli* and *Shigella* decline sharply (Firrman et al., 2022). This acidification not only appears to suppress harmful bacteria. It could also reduce bacterial enzymes that contribute to cholesterol nucleation. This would diminish stone-promoting factors at the source (Zhang et al., 2020).

For hypoxia induction, colonic epithelial cells preferentially utilize butyrate. Its β-oxidation consumes substantial oxygen. This generates an anaerobic environment (Ornelas et al., 2023). This condition inhibits aerobic pathogens such as *Salmonella*. It also supports the survival of beneficial anaerobic bacteria. This would favor a microbiota composition protective against gallstone formation.

SCFAs could also promote the secretion of AMPs to eliminate harmful bacteria directly. SCFAs activate G-protein-coupled receptor 43 (GPR43). This stimulates the release of regenerating islet-derived protein 3γ and β-defensin (Liu T. et al., 2023). Furthermore, SCFAs seem to inhibit histone deacetylase. This would enhance the expression of AMP-related genes. Animal studies have shown that oral sodium butyrate increases defensin expression in mice with intestinal dysbiosis (Fusco et al., 2026). Unlike pH reduction and hypoxia, which primarily exert inhibitory effects, AMPs directly kill pathogens, forming a dual mechanism of suppression and clearance.

With respect to pigment gallstone formation, SCFAs appear to inhibit the pathogens that produce *β*-glucuronidase and phospholipase. Simultaneously, they lower local pH. This would directly inactivate these enzymes. These combined effects could suppress the formation of brown pigment stones (Dan et al., 2023). In summary, by acidifying the intestinal lumen, inducing hypoxia, and promoting AMP secretion, SCFAs could remodel the gut microbiota and reduce pro-lithogenic factors. This might ultimately inhibit cholesterol and brown pigment stone development. This effect is illustrated in Figure 4, panel 4.

### 4.5 Alleviation of cholecystitis

At present, the causal link between cholecystitis and gallstone formation remains unclear. However, some studies have shown that cholecystitis accelerates gallstone development (Yu J. et al., 2024). Gallstones are accompanied by elevated inflammatory factors such as Tumor Necrosis Factor-*α* (TNF-α) (Liu et al., 2025b). It induces the secretion of MUC5AC and MUC2 via the epidermal growth factor receptor pathway. This would facilitate cholesterol crystal nucleation and aggregation (Zhao Z. et al., 2024). Inflammation accelerates gallstone formation. Formed stones further worsen cholecystitis. This creates a vicious cycle. As potential anti-inflammatory regulators, SCFAs could help interrupt this process. They might curb inflammatory factors and reduce the expression of chemokines and adhesion molecules (Wang J. et al., 2026).

SCFAs appear to suppress inflammatory factor production. This effect is primarily mediated through downstream receptors, including GPR109A, GPR43, and GPR41. These receptors are widely distributed in the intestine, liver, and gallbladder. They help maintain barrier integrity, regulate lipid and bile acid metabolism, and limit aberrant mucin hypersecretion. Within the immune system, they also modulate inflammatory mediator release. Specifically, butyrate seems to bind to GPR109A. This would inhibit Nuclear Factor-κB (NF-κB) activation and increase anti-inflammatory Interleukin-10 (IL-10) levels (Singh et al., 2022). It also appears to suppress HDAC3. This could lower macrophage-derived IL-6 and TNF-α. It might weaken their stimulation of mucin secretion (Parada-Venegas et al., 2025). Propionate has been reported to activate GPR43 to inhibit HDAC1 and promote IL-10 release. This could help alleviate cholecystitis (Kang et al., 2024). Additionally, they might act by GPR41 to downregulate the levels of IL-1β and IL-17A (Zhang et al., 2026). Though acetate has no HDAC repressive effect, it appears to activate GPR43. This would reduce monocyte chemoattractant protein-1 (MCP-1) production, which is triggered by TNF-α (Al-Roub et al., 2021). It seems to form complementary anti-inflammatory effects with butyrate and propionate.

Furthermore, SCFAs could regulate chemokines and adhesion molecules. Butyrate and propionate appear to downregulate NF-κB activity. This would reduce the expression of chemokines such as IL-8 and MCP-1. It could also lower the expression of adhesion molecules, including intercellular adhesion molecule-1 and vascular cell adhesion molecule-1. In turn, this might help prevent inflammatory cell infiltration into the gallbladder (Al-Roub et al., 2021).

The alleviation of cholecystitis by SCFAs could also help prevent pigment gallstone formation. This may occur through the modulation of bilirubin metabolism. Inflammation downregulates MRP2. This reduces the biliary secretion of conjugated bilirubin. By suppressing inflammation, SCFAs might promote bilirubin secretion. They could limit the accumulation of unconjugated bilirubin. Inflammation also stimulates the release of endogenous β-glucuronidase from the gallbladder epithelium. The anti-inflammatory actions of SCFAs seem to inhibit this enzyme. This effect would complement the pH-mediated inhibition of bacterial β-glucuronidase. In addition, SCFAs appear to suppress cholecystitis and thereby reduce mucin hypersecretion (Zhao Z. et al., 2024). Meanwhile, SCFAs also seem to help preserve gallbladder contractility. This would relieve bile stasis and reduce calcium salt deposition (Peng et al., 2025). Through these interconnected pathways, SCFAs could contribute to the prevention of pigment stone formation. In summary, SCFAs could help reduce inflammatory factors, chemokines, and adhesion molecules, potentially relieving cholecystitis and slowing cholesterol and pigment stone progression, as shown in Figure 4, panel 5.

## 5 Intervention strategies

Based on the five mechanisms by which SCFAs may inhibit gallstone formation, targeting the gut microbiota and its metabolites, especially SCFAs, has emerged as an area of interest for non-surgical intervention. However, it must be emphasized that the current evidence is almost entirely derived from animal studies. Few SCFA-targeted interventions have been validated for gallstone prevention in general populations. Current methods under exploration include probiotics, prebiotics, postbiotics, and fecal microbiota transplantation (FMT). Their goal is to raise SCFA levels, directly or indirectly, to exert anti-lithogenic effects. Table 5 presents a summary of the relevant literature and future directions.

**Table 5:** Overview of evidence and future directions for SCFA-based interventions against gallstones.

Intervention category	Study type	Subjects/Models	Main results and conclusions	Limitations	Future research directions	References
Probiotics	Randomizd controlled clinical trial	Patients after bariatric surgery	The probiotic preparation containing *Clostridium butyricum* exerted a comparable gallstone preventive effect to ursodeoxycholic acid, with fewer adverse reactions.	Small sample size; short follow-up period; optimal dosage and treatment course remain undetermined.	Screen specific strains with definite anti-lithogenic effects and high safety; conduct large-scale and long-term clinical trials to verify preventive efficacy in high-risk populations.	Han et al. (2022)
Probiotics	Animal experiment	C57BL/6J mice	*Lactobacillus acidophilus* and *Lactobacillus fermentum* reduced the incidence of cholesterol gallstones. The mechanism was associated with the inhibition of hepatic HMGCR and gallbladder mucin expression.	Only two strains were investigated; the experimental duration was short.		Oh et al. (2021)
Probiotics	Animal experiment	C57BL/6J mice	*Lactobacillus taiwanensis* inhibits gallstone formation by elevating lipoxin A4 levels and reducing neutrophil extracellular trap formation.	Mechanistic exploration is insufficient; no verification in human subjects.		Liu et al. (2026)
Prebiotics	Animal experiment	C57BL/6J mice	Fructan completely inhibited cholesterol gallstone formation and improved multiple metabolic indicators.	The study only focused on prevention; only male mice were used.	Carry out dietary fiber intervention trials targeting high-risk populations; develop personalized formulas based on individual gut microbiota profiles.	Liu et al. (2025a)
Prebiotics	Animal experiment	Prairie dogs	Psyllium soluble fiber reduced the gallstone incidence from 80 to 30% and decreased the cholesterol saturation index.	There are interspecies differences between animals and humans; the underlying mechanism remains unclear.		Zhuang et al. (2021)
Prebiotics	*In vitro* simulation	M-SHIME gut model	Soluble corn fiber increased the abundance of *Bifidobacterium* and other microbes, and raised the production of acetate, propionate and butyrate.	The *in vitro* model cannot fully reflect the *in vivo* conditions.		Arroyo et al. (2023)
Postbiotis	Animal experiment	C57BL/6J mice	Sodium butyrate markedly reduced the incidence of cholesterol gallstones from 100 to 25%, via activating the PPAR-γ or FXR signaling pathway.	All relevant studies were performed in animals; clinical evidence in humans is lacking.	Promote the translational research of butyrate from animal experiments to clinical trials; define effective dosage and pharmacokinetic characteristics; develop sustained-release or targeted delivery systems.	Ye et al. (2021)
Postbiotis	Animal experiment	C57BL/6J mice	Sodium butyrate lowered the incidence of cholesterol gallstones and regulated cholesterol metabolism.	Relevant research mainly focused on inflammation; direct observation of gallstone formation was not conducted.		Sun et al. (2024)

### 5.1 Probiotics

In animal models, certain *Lactobacillus* strains have been shown to reduce the incidence of gallstones induced by a lithogenic diet (Oh et al., 2021; Liu et al., 2026). In humans, evidence is limited to a single clinical trial. The study showed that a probiotic preparation containing *Clostridium butyricum* was not inferior to ursodeoxycholic acid in preventing gallstones among post-bariatric surgery patients. It was also associated with fewer adverse reactions (Han et al., 2022). However, this trial was conducted in a highly specific population, and the sample size was not explicitly reported, limiting the generalizability of its findings. Probiotic interventions also face several limitations, including the risk of antibiotic resistance gene transfer from live bacteria, potential infection risks in immunocompromised individuals, and strain-specific effects with a lack of standardization (Fang et al., 2024; Wang et al., 2025). Regulatory frameworks for probiotics as therapeutic agents vary considerably across jurisdictions, presenting an additional barrier to clinical translation.

### 5.2 Prebiotics

Preclinical studies have explored whether prebiotics may indirectly inhibit gallstones by selectively promoting the growth of SCFA-producing bacteria. *In vitro* gut microbiota models have shown that soluble corn fiber can increase the abundance of SCFA producers such as *Bifidobacterium* and raise the production of acetate, propionate, and butyrate (Arroyo et al., 2023). In rodent models, it has been reported that soluble dietary fiber, such as psyllium, was associated with a reduced incidence of gallstones (Zhuang et al., 2021). Fructooligosaccharides and resistant starch have been reported to display protective effects in lithogenic diet-induced models, with the former even achieving complete inhibition. However, this result may be attributable to the small number of experimental animals used (Yan et al., 2024; Liu et al., 2025a). Whether these preclinical observations can be extrapolated to humans remains uncertain, as direct validation from large-scale clinical trials is lacking. Furthermore, individual differences in gut microbiota may lead to variable responses, and high doses may cause tolerability issues such as bloating.

### 5.3 Postbiotics

Postbiotics are nonliving microorganisms or their components and metabolites. They could avoid the safety risks associated with live bacteria, thus offering theoretical advantages for clinical translation (Fang et al., 2024). Among them, sodium butyrate is the most representative SCFA postbiotic. It has shown potential to inhibit gallstone formation in multiple animal studies. Long-term administration of sodium butyrate significantly reduced gallstone incidence in mice fed a lithogenic diet (Ye et al., 2021; Sun et al., 2024). It also alleviated calculous cholecystitis (Chen et al., 2024). However, these effects are currently based solely on preclinical observations, with a complete absence of human studies, and the actual clinical benefit remains unknown. It should also be noted that not all SCFAs produce beneficial effects, as high concentrations of acetate may promote ghrelin secretion, while certain propionate derivatives may interfere with insulin signaling (Fang et al., 2024). This raises a deeper question. It is unclear whether SCFA-mediated anti-lithogenic effects follow a linear dose–response pattern or exhibit an optimal therapeutic window. Most animal studies have used supraphysiological doses that may not reflect the safe and effective range in humans. This issue is further complicated by substantial population heterogeneity. Differences in gut microbiota composition, dietary habits, and host genetic factors could lead to highly variable responses. The same postbiotic regimen may produce different outcomes across individuals or populations.

### 5.4 Fecal microbiota transplantation

FMT theoretically provides a comprehensive solution for gallstone treatment by reconstructing the gut microecology. Existing liver disease studies have shown that this technique can increase the abundance of SCFA-producing bacteria, improve bile acid metabolism, and enhance intestinal barrier function (Ma et al., 2025; Saha and Schnabl, 2025). In the gallstone field, only a few studies have hinted that FMT can enhance gut microbial diversity and improve bile acid metabolism in gallstone patients (Tan et al., 2025). However, these findings are indirect or incidental. There is no randomized controlled trial specifically targeting gallstones. This technique also carries clear risks, including infection transmission, donor microbiota variability, and immune-related adverse reactions (Ma et al., 2025). Furthermore, FMT therapies face complex and evolving regulatory requirements. These requirements vary across different jurisdictions. A harmonized framework for quality control, efficacy standards, and post-market surveillance is lacking. This presents additional barriers to clinical translation.

### 5.5 Future research directions

SCFA-based intervention strategies offer a diversified non-surgical avenue for gallstone prevention and treatment. From probiotics and prebiotics to postbiotics and FMT, these strategies cover multiple levels, including live bacteria modulation, substrate promotion, metabolite supplementation, and microbiota reconstruction. This may allow flexible choices according to different populations and clinical needs. Among them, nonliving postbiotics show some safety advantages. Probiotics have demonstrated preliminary gallstone prevention effects in some clinical studies with relatively few adverse reactions. Although FMT is still in early exploration, it theoretically has comprehensive regulatory advantages by reconstructing the whole gut microecology. Overall, this strategy system provides some new ideas for the prevention and treatment of gallstones.

Nevertheless, studies on the above intervention strategies still have certain limitations. Future research should advance in several interconnected directions. Large-scale, long-term, multicenter clinical trials are needed to verify the efficacy and safety of SCFA-producing postbiotics in the primary and secondary prevention of gallstones. In parallel, multi-omics and artificial intelligence can be harnessed. They can be used to construct individualized prediction models based on the microbiota-metabolism-gene axis, thus enabling precision intervention. Another promising direction is the development of specific SCFA receptor agonists. Designing sustained-release butyrate formulations is another possibility. These strategies could potentially circumvent live-bacteria-related risks and improve pharmacokinetics. Furthermore, standardized donor screening and regulatory frameworks are essential for the FMT field. Such measures would allow cautious exploration of FMT application in refractory gallstones under rigorous risk control.

## 6 Limitations of current evidence

Before translating the above strategies into clinical practice, several important limitations in the current evidence base must be acknowledged. First, no prospective cohort studies have directly measured SCFA levels and tracked subsequent gallstone incidence. The available human data are almost entirely cross-sectional. This makes it impossible to determine whether altered SCFA levels precede gallstone formation or merely reflect disease-related changes. Moreover, isolating the specific contribution of SCFAs from that of other microbial metabolites is inherently difficult. The gut microbiota produces many bioactive molecules that co-vary with SCFA production. Most interventions that raise SCFA levels also alter other microbial products. Consequently, it is unclear whether the observed anti-lithogenic effects stem solely from SCFAs or from SCFAs together with co-varying metabolites. Furthermore, randomized controlled trials directly testing SCFA-based interventions for gallstone prevention are lacking. The available clinical evidence is very limited. It is often confined to specific populations. Finally, a clear distinction must be drawn between statistical association and biological causation. Gallstone patients show a reduced abundance of SCFA-producing bacteria. Mendelian randomization studies point to statistical associations. However, these data alone do not establish that SCFAs directly inhibit gallstone formation in humans. Overall, convergent evidence from three sources is needed. These include prospective cohort studies, experimental models that directly verify SCFA effects, and human intervention trials. Only then can the preventive role of SCFAs be considered established.

## 7 Conclusion

This review integrated and summarized the metabolic processes of SCFA-producing bacteria, their association with gallstones, and the potential anti-lithogenic mechanisms of SCFAs. The available evidence comes mainly from preclinical studies and does not establish a definitive causal chain. Nevertheless, it suggests that SCFAs may exert a protective effect against gallstone formation. Based on these mechanisms, this review discussed non-surgical intervention strategies including probiotics and prebiotics, and analyzed their limitations and future research directions. However, a critical uncertainty remains: the direction of causality. Reduced SCFA production may contribute to gallstone formation, but it is equally possible that gallstone disease itself disrupts the gut microbiota through chronic inflammation, bile stasis, or altered dietary patterns, thereby secondarily reducing SCFA levels. Resolving this bidirectional relationship represents a central challenge for the field and limits the clinical translation of related strategies. Several approaches may help distinguish causality. Mendelian randomization studies can provide genetic evidence, longitudinal prospective studies can track changes over time, and pre- and post-intervention observations in gallstone patients can reveal dynamic responses. Together, these methods offer evidence from different angles and could gradually resolve this central challenge.

## Glossary

SCFAs: Short-chain fatty acids
BUT: Butyryl-CoA:acetate CoA-transferase
HBD: 3-hydroxybutyryl-CoA dehydrogenase
BCD: Butyryl-CoA dehydrogenase
1,2-PD: 1,2-propanediol
PduCDE: Cobalamin-dependent glycerol/diol dehydratase
Pyr: Pyruvate
Suc: Succinate
BSH: Bile salt hydrolase
FXR: Farnesoid X receptor
FGF15: Fibroblast growth factor 15
HMGCR: 3-hydroxy-3-methylglutaryl coenzyme A reductase
ABCG5/8: ATP-binding cassette subfamily G member 5/8
LPS: Lipopolysaccharide
MUC5AC: Mucin 5 AC
MUC2: Mucin 2
ZO-1: Zonula occludens-1
HIF-1*α*: Hypoxia-inducible factor-1α
AMP: Antimicrobial peptide
GPR43: G-protein-coupled receptor 43
TNF-α: Tumor necrosis factor-α
NF-κB: Nuclear factor-κB
IL-10: Interleukin-10
MCP-1: Monocyte chemoattractant protein-1
FMT: Fecal microbiota transplantation
